# Advances in RNA cytosine-5 methylation: detection, regulatory mechanisms, biological functions and links to cancer

**DOI:** 10.1186/s40364-020-00225-0

**Published:** 2020-09-14

**Authors:** Chen Xue, Yalei Zhao, Lanjuan Li

**Affiliations:** 1 State Key Laboratory for Diagnosis and Treatment of Infectious Diseases, The First Affiliated Hospital, College of Medicine, Zhejiang University, No. 79 Qingchun Road, Shangcheng District, Hangzhou, 310003 Zhejiang China; 2 National Clinical Research Center for Infectious Diseases, The First Affiliated Hospital, College of Medicine, Zhejiang University, Hangzhou, 310003 China; 3 Collaborative Innovation Center for Diagnosis and Treatment of Infectious Diseases, The First Affiliated Hospital, College of Medicine, Zhejiang University, Hangzhou, 310003 China

**Keywords:** 5-methylcytosine, RNA modification, Epitranscriptome, Detection techniques, Biological functions

## Abstract

As an important posttranscriptional modification of RNA, 5-methylcytosine (m^5^C) has attracted increasing interest recently, with accumulating evidence suggesting the involvement of RNA m^5^C modification in multiple cellular processes as well as tumorigenesis. Cooperatively, advances in m^5^C detection techniques have enabled transcriptome mapping of RNA methylation at single-nucleotide resolution, thus stimulating m^5^C-based investigations. In this review, we summarize currently available approaches for detecting m^5^C distribution in RNA as well as the advantages and disadvantages of these techniques. Moreover, we elucidate the regulatory mechanisms of RNA m^5^C modification by introducing the molecular structure, catalytic substrates, cellular distributions and biological functions of RNA m^5^C regulators. The functional consequences of m^5^C modification on mRNAs, tRNAs, rRNAs and other RNA species, including viral RNAs and vault RNAs, are also discussed. Finally, we review the role of RNA m^5^C modification in cancer pathogenesis and progression, in hopes of providing new insights into cancer treatment.

## Background

Epigenetic modifications, mainly including DNA methylation and histone modifications, are chemical alterations in nucleic acids that do not change the DNA sequence but play key roles in heredity, growth, longevity, aging and disease [1, 2]. DNA 5-methylcytosine (m^5^C) has been found to be the most abundant DNA modification in mammalian cells and is characterized by the addition of a methyl group at the carbon-5 position of the cytosine base [3]. In eukaryotes, the role of m^5^C in DNA and its oxidized derivatives, including 5-hydroxymethylcytosine (5hmC), 5-formylcytosine (5fC), and 5-carboxylcytosine (5caC), has been extensively studied (reviewed in [4]). In recent years, great progress has been made in the research of RNA modifications, which were originally regarded as fine-tuning chemostructural features of non-protein-coding RNAs but are now considered to be dynamically regulated, reversible and widespread posttranscriptional regulators in diverse cellular processes (reviewed in [5]). Predominant mammalian RNA methylation modifications include N6-methyladenosine (m^6^A), N1-methyladenosine (m^1^A), pseudouridine (Ψ) and m^5^C, but previous studies on RNA methylation have focused on m^6^A [5, 6]; however, emerging evidence has gradually uncovered the role of RNA m^5^C in posttranscriptional regulation.

With the advances in RNA m^5^C detection techniques, including bisulfite sequencing, m^5^C RNA immunoprecipitation sequencing (m^5^C-RIP-seq), 5-AZAcytidine-mediated RNA immunoprecipitation sequencing (Aza-IP-seq) and methylation-individual nucleotide resolution crosslinking immunoprecipitation sequencing (miCLIP-seq), over 10,000 potential sites of m^5^C modification were detected within the whole human transcriptome [7], and the existence of m^5^C was found in diverse RNA species from various organisms, not only in ribosomal RNA (rRNA), messenger RNA (mRNA) and transfer RNA (tRNA) but also in viral RNA, vault RNA, small nuclear RNA, small nucleolar RNA, pseudogenes, natural antisense transcripts, enhancer RNA, long noncoding RNA (lncRNA) and microRNA (miRNA) [8–15]. The distribution of m^5^C differs among different organisms, as it has been common to find m^5^C methylation substrates in the tRNA and mRNA of eukaryotes and archaea, while no m^5^C methylation substrates have been detected in bacteria [16]. It has been demonstrated that RNA m^5^C is regulated by “writers”, “erasers”, and “readers”, referring to RNA m^5^C methyltransferases (RCMTs), which are enzymes of the ten-eleven translocation (TET) family and RNA binding proteins [16]. The alteration of m^5^C modification as well as its regulators has been proven to influence RNA stability, gene expression, and protein synthesis and thus has a great impact on various cellular and physical processes [15, 17–22]. Additionally, to date, RNA m^5^C modification and its regulators have been confirmed to play crucial roles in the pathogenesis of bladder cancer [23], hepatocellular carcinoma (HCC) [24], glioblastoma multiforme (GBM) [14] and leukemia [18], indicating the promising prospect of m^5^C modification in cancer treatment.

Herein, we introduce the principles of m^5^C mapping methods as well as their advantages and disadvantages. Next, we summarize the mechanisms and biological functions of m^5^C modification of RNAs. Finally, by discussing the role of m^5^C in tumorigenesis and cancer progression, we propose m^5^C-based approaches for cancer diagnosis, prognosis and clinical treatment. Although some statements in our study may briefly touch on other reviews [8, 16, 25], which discussed the detection methods and biological functions of RNA m^5^C methylation in depth, we focus on literature published in the past five years and are the first to thoroughly discuss the clinical prospect of RNA m^5^C in cancer treatment.

## Approaches in detecting m^5^C distribution in RNA

Although m^5^C was discovered in RNA almost 60 years ago, little progress has been made regarding the distribution and biological roles of RNA m^5^C due to a lack of reliable and sensitive methods for RNA methylation detection [26]. With the development of next-generation sequencing (NGS) techniques, RNA methylation can be mapped transcriptome-wide at single-nucleotide resolution. Herein, we summarize the principles of currently available approaches for the characterization of RNA m^5^C patterns, including bisulfite sequencing, m^5^C-RIP-seq, Aza-IP-seq and miCLIP-seq. Additionally, their advantages and limitations are discussed to provide a more comprehensive view of RNA m^5^C mapping (Table 1).

**Table 1:** Approaches for the mapping of m5C in RNA

Techniques	Principle	Advantages	Disadvantages	Nucleotide Resolution
Bisulfite sequencing	Unmethylated Cs are deaminated to Us in the presence of sodium bisulfite, while methylated Cs remain unchanged	Unbiased transcriptome-wide mapping at single nucleotide resolution and with high specificity	Unable to detect m^5^C sites in low-abundance RNAs, and fail to distinguish m^5^C from other types of cytosine modifications	Single nucleotide resolution
RIP-seq	RNA immunoprecipitation	Specific mapping of m^5^C sites in low abundance RNAs	Not at single nucleotide resolution	100–150 nt
Aza-IP-seq	Protein immunoprecipitation	Investigate specific catalytic sites of RCTMs	Missing of unstably converted m^5^C sites, random incorporation of cytidine analogue to DNAs, and toxicity to cells	Enzyme-specific nucleotide resolution
miCLIP-seq	Protein immunoprecipitation	Investigate specific catalytic sites of RCTMs	Time-consuming and costly	Enzyme-specific nucleotide resolution

## Bisulfite sequencing

Sodium bisulfite has been applied as a robust and reliable method for DNA m^5^C analysis since the 1990s [27]. In an acidic pH, sodium bisulfite reacts with methylated and unmethylated C, resulting in deamination to uracil sulfonate/5-methyluracil sulfonate, which can be further converted to uracil/thymine at basic pH. It was not originally considered an available technique for RNA methylation detection, as the stability of phosphodiester bonds in RNA might be disrupted due to harsh chemical and temperature treatment, and the methylation status of individual RNA sequences failed to be properly interrogated [28]. In 2009, a versatile bisulfite deamination protocol for RNA m^5^C detection was developed based on PCR that allowed amplification of cDNA from low-abundance cellular RNAs [29]. In the presence of sodium bisulfite, unmethylated cytosines (Cs) can be chemically deaminated to uracils (Us), which are ultimately replaced by thymines (Ts) during subsequent PCR amplification, while methylated Cs remain unchanged in this process; following this mechanism, bisulfite sequencing is designed to differentiate unmethylated and methylated Cs and thus investigate the prevalence of cytosine methylation in RNA [29]. Coupled with NGS techniques, bisulfite conversion is now regarded as the gold standard for mapping modified cytosine in RNA at single nucleotide resolution and with high specificity. However, the broader application of bisulfite sequencing is hindered by the degradation of low-abundance RNAs during the conversion process [30]. Additionally, it fails to distinguish m^5^C from other types of cytosine modifications, such as 5hmC [31]. Further efforts should be made to optimize the validity and scope of bisulfite sequencing.

## m^5^C-RIP-seq

Originally applied to m^6^A detection in randomly fragmented RNA transcripts [32], m^5^C-RIP was later adapted to detect RNA m^5^C in bacterial, archaeal, yeast and plant transcriptomes [33]. Conducted with a m^5^C-specific antibody combined with a deep sequencing technique, this transcriptome-wide protocol is available for mapping m^5^C sites in low-abundance RNAs; it has high specificity and no harsh reaction conditions, as presented in bisulfite sequencing. However, as the antibody and sequencing method cannot detect modifications with single-nucleotide resolution, as the sequence read by antibodies is usually 100–150 nt in length [30]. Given that bisulfite sequencing cannot distinguish m5C from other types of cytosine modifications, such as 5hmC, m5C-RIP-seq can be used to confirm the methylation of target RNA recognized by bisulfite to improve the stability and accuracy of bisulfite sequencing.

## Aza-IP-seq and miCLIP-seq

Aza-IP-seq and miCLIP-seq are designed to investigate specific catalytic sites of RCMTs by immunoprecipitation. All characterized mammalian RCMTs form a reversible covalent intermediate with their cytosine substrate—a covalent linkage that is trapped when formed with the cytosine analog 5-AZAcytidine (5-AZA-C) [34]. Replaced by the cytidine analog 5-AZA-C, an RNA m^5^C site traps its target methyltransferase by forming a covalent enzyme-RNA adduct, which is then immunoprecipitated with enzyme- or tag-targeted antibodies. During reverse transcription and sequencing, the covalent bond between RNA and methyltransferase is broken, and the release of RNA triggers a hydrolytic opening of the 5-AZA-C ring, which is later read as a guanine (G). Not surprisingly, Aza-IP enables identification of RCMT-specific m5C sites at single nucleotide resolution. However, it is also limited for some reasons, including the toxicity of 5-AZAcytidine to cells, the possible absence of RNAs that are not stably replaced by 5-AZA-C, and random incorporation of cytidine analog into DNAs [35]. Remarkably, a cytosine-to-guanine (C → G) transversion occurs specifically at target cytosines, allowing the simultaneous identification of the precise target cytosine within each RNA.

In contrast to Aza-IP, due to the mutation of the conserved cysteine at the catalytic domain (C271A) of the RNA methyltransferase, an irreversible covalent bond is formed between the enzyme and catalytic site during the miCLIP process [36]. Following immunoprecipitation and deep sequencing, m^5^C sites are mapped at the + 1 site of the sequencing reads, which is distinct from the C-to-G transversion signatures in Aza-IP. Despite its high specificity, miCLIP requires a large amount of time and money.

## RNA m^5^C regulatory mechanisms

RNA 5-cytosine methylation is a dynamic and reversible process modified by 3 major regulators, referred to as writers, erasers and readers. RCMTs, which include NOL1/NOP2/sun (NSUN) subgroups of methyltransferases and DNA methyltransferase homologue DNMT2, are recognized as writers for catalyzing cytosine-5 methylation [37]. Additionally, RNA m^5^C binding proteins (readers), such as ALYREF and YBX1, exert biological effects by recognizing and binding to m^5^C sites [24]. The eraser protein demethylases, including enzymes of the TET family, exhibit reversible effects by mediating written RNA degradation. Herein, we summarize the RNA m^5^C regulators in detail, emphasizing their molecular structures, catalytic substrates, cellular distributions and biological functions (Table 2).

**Table 2:** Summary of RNA m^5^C regulators

Regulator		Molecular structure	Catalytic substrate	Cellular distribution	Cellular processes involved
Writer	NSUN2	A SAM binding site and a catalytic domain, which contains two cysteine residues	tRNA at position C34, 40, 48–50, mRNA (near the start codons and stop codons in coding sequence, also in untranslated region), rRNA, viral RNA, vault RNA	G1 phase: nucleolus, S phase: between nucleolus and nucleoplasm, G2 phase: cytoplasm, M phase: centrioles	Root development of plants, mitochondrial oxidative phosphorylation, protein synthesis, cell cycle progression, HIV replication, Epstein-Barr virus degradation, epidermal differentiation and tumorigenesis
	NUSN1		25 s rRNA at position C2870 in domain V	Predominantly nucleolus and weaker cytoplasmic staining	Tumor aggressiveness, cell cycle progression, chromatins organization and HIV-1 latency
	NSUN4		12S rRNA at position C911	Mitochondria	Tumorigenesis
	NUUN5		25 s rRNA at position C2278 in domain IV	Nucleolus	Cell senescence and stress response
	NSUN3		mt-tRNA^Met^ at wobble base C34	Mitochondria	Mitochondrial oxidative phosphorylation, embryonic stem cell differentiation
	NSUN6		tRNA^Cys^ and tRNA^Thr^ at position C72	Golgi apparatus and pericentriolar matrix	tRNA biogenesis
	DNMT2	A SAM binding site and a catalytic domain, which contains one cysteine residue	tRNA^Asp-GTC^, tRNA^Gly-GCC^ and tRNA^Val-AAC^ at position C38, mRNA	Cytoplasm	Tumorigenesis, protein synthesis, cell differentiation, malarial parasite pathogenicity and HIV-1 RNA survival
Eraser	TET1	–	Coding and non-coding RNAs	Nucleus	5-methylcytidine oxidation
	TET2	–		Nucleus	
	TET3	–		Nucleus and cytoplasm	
Reader	ALYREF	–	mRNA and retroviral RNA	Nucleus	mRNA nuclear-cytoplasmic shuttling, viral RNA export and replication
	YBX1	A cold-shock domain	mRNA	Cytoplasm	mRNA stabilization, embryogenesis, tumorigenesis

## Writers

The DNA methyltransferase homologue DNMT2 contains a Rossman-fold catalytic domain and an S-adenosyl methionine (SAM) binding site. DNMT2 and NSUN2 have complementary target specificities [38]. DNMT2 mainly mediates the m5C modification of tRNA. Studies have confirmed that after knocking out DNMT2, there is no detectable change in mRNA levels, but the steady-state level of unmethylated tRNAs is significantly reduced, resulting in a significant decrease in the protein synthesis rate. These findings indicate that the complete loss of cytosine-5-tRNA methylation has a significant impact on the stability of certain tRNAs and establishes the role of tRNA methylation in protein translation [39].

Similar to DNMT2, the NSUN family of proteins was also found to contain a Rossman-fold catalytic domain and a SAM binding site. Using SAM as a methyl group donor, RCMTs covalently bind with the RNA cytosine pyrimidine ring through the conserved cysteine residue and catalyze 5-cytosine methylation. Structurally, the NSUN family of proteins contains two cysteine residues in motif IV and motif VI within the catalytic domain [40]. The thiol group of the cysteine in motif VI, together with threonine (TC), forms a conserved dipeptide motif to catalyze the covalent binding between the enzyme and target cytosine, thus promoting the methylation of the carbon-5 atom by electrophilic SAM [17]. Continually, the dipeptide motif formed by cysteine in motif IV and proline (PC) catalyzes covalent bond breakage as well as the release of methylated RNA and enzymes.

The NSUN family of proteins comprises 7 members, including NSUN1, NSUN2, NSUN3, NSUN4, NSUN5, NSUN6 and NSUN7, among which NSUN2 was the first-discovered and is the most well-elucidated member. Dynamic cellular locations of NSUN2 were demonstrated to be associated with cell cycle progression of human epidermal cells, and it was observed to be enriched in the nucleolus in G1, move to the position between nucleoli and nucleoplasm during S phase, and localize to cytoplasm in G2 and centrioles during M phase [41]. Moreover, recent evidence has also shown the mapping of m^5^C in mitochondrial transfer RNAs (mt-tRNAs) at position C48–50 within mammalian mitochondria [42]. Originally identified to methylate cytoplasmic tRNAs at the junction of the variable loop and the T stem spanning positions 34, 40 and 48–50, NSUN2 has gradually been found to have diverse RNA substrates, including rRNAs, tRNAs, mRNAs, mt-tRNAs and viral RNAs [9–11, 43–45]. Additionally, NSUN2 exerts multiple functions in the regulation of plant root development, mitochondrial oxidative phosphorylation, protein synthesis and cell cycle progression in response to oxidative stress stimuli [46], and it has also been reported to regulate HIV replication [44], Epstein-Barr virus degradation [45], epidermal differentiation [47] and tumorigenesis [23, 48, 49]. In addition, studies have shown that NSUN2 plays a role in spindle assembly during mitosis and chromosome segregation. A conserved residue of this gene undergoes a missense change in autosomal recessive mental retardation, causing NSUN2 to fail to localize to the cerebellar Purkinje cell nucleus. Rennai showed that the involvement of NSUN2 highlights the role of RNA methyltransferase in human neurocognitive development [50]. NSUN1, NSUN4 and NSUN5 have been confirmed to play crucial roles in rRNA methylation, contributing to ribosome biogenesis and assembly. In the 1990s, NSUN1 was demonstrated to be involved in 60S ribosomal subunit biogenesis and the inhibition of tumor progression. Predominantly localized to nucleoli, Nop2, the yeast homologue of NSUN1, was reported to catalyze 25S rRNA methylation at position C2870 in domain V, thus affecting 60S large ribosome subunit biogenesis [12]. Recent studies have indicated the role of NSUN1 as a multifunctional protein that is crucially associated with RNA modification, tumor aggressiveness [51], cell cycle progression [52], chromatin organization [18] and HIV-1 latency [53]. NSUN4 is also identified as a rRNA-specific RCMT and is transported into mitochondria in an N-terminal 26-amino acid motif-dependent manner [54]. By interacting with the mitochondrial regulatory factor MTERF4, NSUN4 is recruited to the large subunit of the mitochondrial ribosome, thus facilitating mitochondrial ribosome assembly by modifying 12S rRNA methylation at position C911 [54]. Notably, NSUN4 is now considered an essential m^5^C regulator that is highly involved in HCC progression [24]. Another rRNA-specific RCMT is NSUN5 (also called Rcm1 in yeast), which is localized to nucleoli, and the methylation substrate of NSUN5 is at C2278 within the conserved domain IV of 25S rRNA [55]. The knockout of NSUN5 results in the extension of life span and increased resistance to multiple stresses in yeast, worms and files, indicating the important role of NSUN5 in cell senescence and stress response [56].

RCMTs exert broad regulation not only of rRNAs but also of tRNAs, which are predominantly modified by NSUN3, NSUN6 and DNMT2. NSUN3 is localized to mitochondria and is required for the methylation of mitochondrially encoded transfer RNA methionine (mt-tRNA^Met^) at the “wobble base C34” within the anticodon loop, and the methylated cytosine is further oxidized to 5fC for normal mitochondrial translation of the respiratory chain complex and oxidative phosphorylation. The mutation of NSUN3 is tightly linked to mitochondrial disease and skewed embryonic stem cell differentiation towards the meso- and endoderm lineages instead of neuroectoderm [57, 58]. Another tRNA methylation regulator is NSUN6, which partially resides in the Golgi apparatus and pericentriolar matrix in the cytoplasm and mediates specific methylation on tRNA^Cys^ and tRNA^Thr^ at position C72, which affects tRNAs in a late step in their biogenesis. Both tRNA substrates share a 3′-CCA sequence in the structure, which can be recognized by NSUN6 to carry out cytosine methylation [59]. Distinct from other RCMTs, DNMT2, which was renamed tRNA methyltransferase 1 (TRMDT1), was previously classified as a DNA methyltransferase, as it has the typical sequence and structural features of a DNA methyltransferase [60]. However, further investigations indicated that DNMT2 acts more like an RCMT, since the enrichment of DNMT2 was observed in the cytoplasm and since knockdown of DNMT2 in mice failed to change the DNA methylation level [61]. The reported tRNA substrates of DNMT2 are tRNA^Asp-GTC^, tRNA^Gly-GCC^ and tRNA^Val-AAC^, all of which contain 3′-GCG and 5′-CA sequences around the methylated C38 position within the anticodon loop [39]. A recent study showed that DNMT2 depletion led to a decrease in the m^5^C methylation rate in mRNA, indicating that DNMT2 also catalyzes the methylation of other RNA types aside from tRNA [13]. Similar to NSUN2, DNMT2 is also a multifunctional enzyme that broadly participates in tumorigenesis [13], protein synthesis [62], cell differentiation [63], malarial parasite pathogenicity [64] and HIV-1 RNA survival [65].

## Erasers

Enzymes of the TET family (TET1, UniProtKB Q8NFU7; TET2, UniProtKB Q6N021; TET3, UniProtKB O43151) are Fe (II)- and α-ketoglutarate (α-KG)-dependent dioxygenases and were initially found to catalyze the oxidation of DNA 5-methyl-2′-deoxycytidine (5mdC) to form 5-hydroxymethyl-2′-deoxycytidine (5hmdC), 5-formyl-2′-deoxycytidine (5fdC), and 5-carboxyl-2′-deoxycytidine (5cadC) [66]. Members of the TET family have distinct cellular distributions, and it was reported that TET3 is located in the nucleus and cytoplasm, while TET1 and TET2 are primarily detected in the nucleus [67]. Recently, the TET family of enzymes has also been found to exhibit activity on RNA 5-methylcytidine (5mrC), and oxidized analogs of 5mrC, including 5-hydroxymethylcytidine (5hmrC), 5-formylcytidine (5frC) and 5-carboxycytidine (5carC), were detected in both coding and noncoding RNAs [68]. It was demonstrated that with the preference for dsDNA, demethylases were tolerant to diverse nucleic acid substrates including dsDNA, ssDNA, ssRNA and DNA-RNA hybrids, and questions remain open regarding how to enhance the specificity and selectivity for TET-mediated oxidation and regarding their structures and biological functions.

## Readers

Aly/REF export factor (ALYREF), the THO subcomplex and the RNA helicase UAP56, are the 3 major components of the transcription-export complex, which plays an essential role in mRNA nuclear export [19]. Mechanistically, the mRNA export adaptor ALYREF is recruited and binds to the 5′ end region of mature RNA in a CBP80-dependent manner as well as to the 3′ end region in a PABPN1-dependent manner, and by directly interacting with the 3′ processing factor CstF64, the overall binding of ALYREF and mRNA is enhanced [9, 19, 20]. Intriguingly, ALYREF protein shows a clear enrichment in mRNA and retroviral RNA m^5^C sites [9, 20]. In human HeLa cells and multiple mouse tissues, ALYREF directly binds to mRNA m^5^C sites and facilitates mRNA nuclear-cytoplasmic shuttling, and the RNA-ALYREF binding affinity as well as the export process are mediated by NSUN2 [9]. In addition, an interaction between ALYREF and methylated retroviral RNA transcripts was observed, and ALYREF was confirmed to play an important role in promoting viral RNA export and replication [20]. However, the molecular structure of the binding adduct and the regulatory mechanisms of ALYREF on RNA m^5^C remain largely unknown; thus, further investigations are urgently needed.

In addition to ALYREF, Y-box binding protein 1 (YBX1) is a newly discovered m^5^C reader protein that was confirmed to regulate mRNA stability in the cytoplasm [23, 69]. In human bladder cancer, YBX1 recognizes and binds with m^5^C-modified mRNAs through an indole ring of W65 within its cold-shock domain (CDS) [23]. Then, by recruiting YBX1-interacting partner ELAV-like protein 1 (ELAVL1), YBX1 regulates mRNA metabolism through the stabilization of m^5^C-modified mRNAs. Similarly, it was also reported in zebrafish that YBX1 contains a crucial residue, Trp45, in its CDS, through which it can recognize and bind to m^5^C-modified mRNAs via π-π interactions, thus stabilizing mRNA by recruiting poly(A)-binding protein cytoplasmic 1a [69].

## Functional consequences of m^5^C modification on different RNA species

Posttranscriptional methylation of 5-cytosine was previously mapped only in tRNAs and rRNAs; however, with the development of high-throughput sequencing technology, m^5^C is now regarded to broadly occur in mRNAs as well as other noncoding RNAs, including viral RNAs, vault RNAs, lncRNAs and microRNAs. Herein, we summarize the functional consequences of m^5^C modification on coding and noncoding RNAs, elucidating the underlying mechanisms and providing a novel view for further investigations.

## mRNA

The regulatory effects of m^5^C modifications on mRNA metabolism have recently become a prevalent issue because of the high abundance of m^5^C sites in mRNAs. The functional role of m^5^C regulation on mRNA can be classified into 3 aspects, which refer to the influence on mRNA translation, transport and stability.

## mRNA translation

**Fig. 1:**
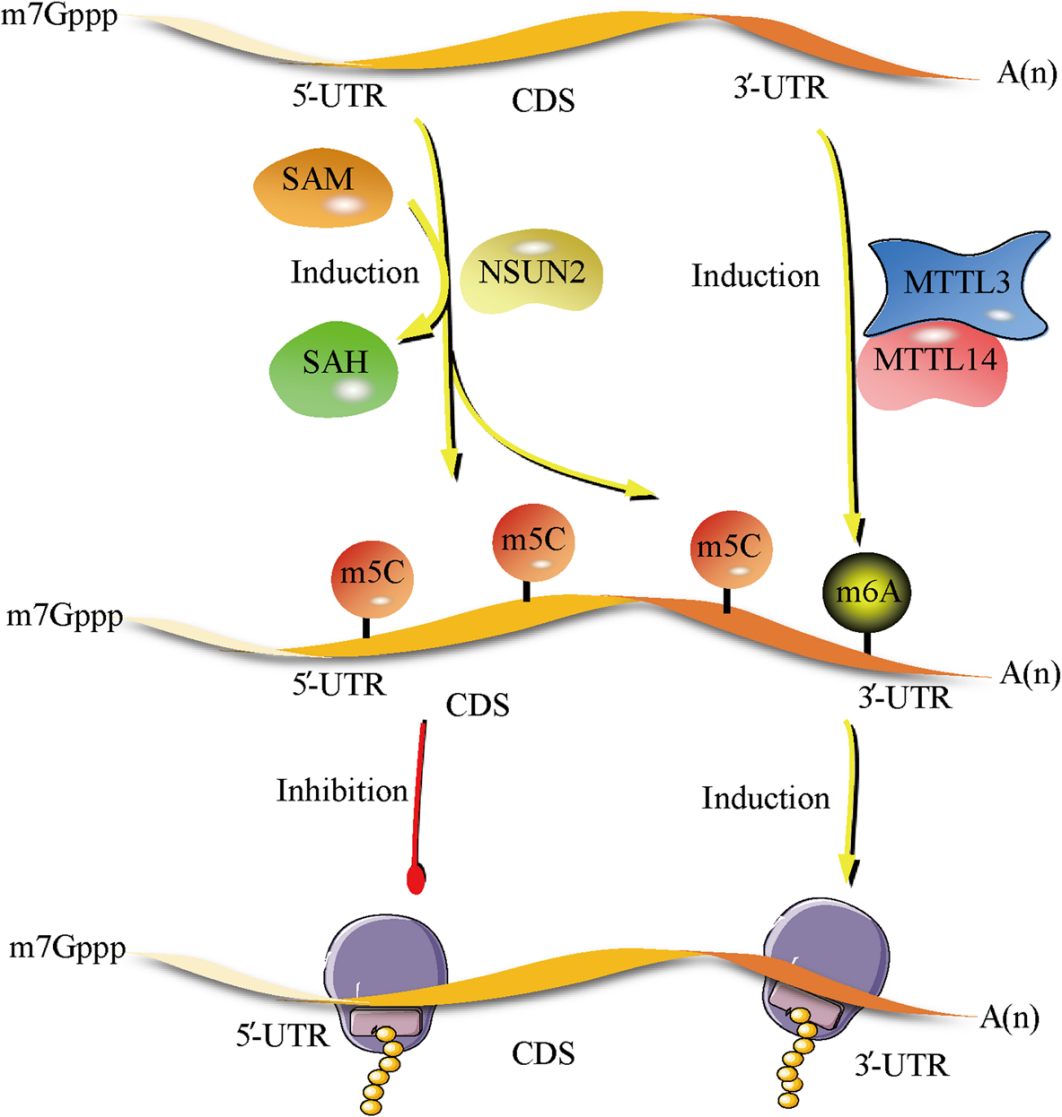
Using SAM as a methyl group donor, NSUN2 catalyzes cytosine-5 methylation in the CDS and 3′-UTR of mRNA. The enrichment of m^5^C deposition in the CDS inhibits mRNA translation efficiency. In contrast, by cooperating with m^6^A methylation, which is modulated by MTTL3/MTTL14, m5C methylation in the 3′-UTR of mRNA promotes mRNA translation and protein synthesis. SAM: S-adenosyl-methionine, SAH: S-adenosyl-homocysteine

The effect of m^5^C modification on mRNA translation is variable due to distinct the methylation profiles of mRNAs (Fig. 1). In *Arabidopsis thaliana*, m^5^C-RIP-seq analysis showed that m^5^C was predominantly enriched in the coding sequence (CDS), and the sequences located immediately after start codons and before stop codons seemed to have peak m^5^C modifications [30]. Further investigations suggested that a high abundance of m^5^C in the CDS was negatively associated with mRNA translation efficiency, as the results showed that a high m^5^C level was correlated with the presence of ribosomal subunits (40S and 60S) and monosomes (80S), which strongly impaired mRNA translation efficiency [30]. Similarly, it was also shown in the HeLa cell transcriptome that candidate m^5^C site contents in the CDS were negatively correlated with mRNA translation efficiency [21]. However, another report based on TP53-deficient human colon carcinoma cells (HCT116 p53−/−) and HeLa cells exhibited opposite results [70]. NSUN2-induced m^5^C methylation, together with methyltransferase like protein 3 (METTL3)/METTL14-induced m^6^A methylation, synergistically mediated p21 mRNA methylation in the 3′-UTR and facilitated p21 mRNA translation efficiency. Furthermore, the increase in p21 expression profoundly affected the outcome of oxidative stress-induced cellular senescence [70]. The contradiction may be related to distinct m^5^C profiles, as a recent study showed that the negative correlation between m^5^C modification and mRNA translation efficiency was significantly associated with methylation site enrichment in the CDS, whereas enhanced methylation in the 3′-UTR was associated with a positive correlation between these features [71].

## mRNA transport

**Fig. 2:**
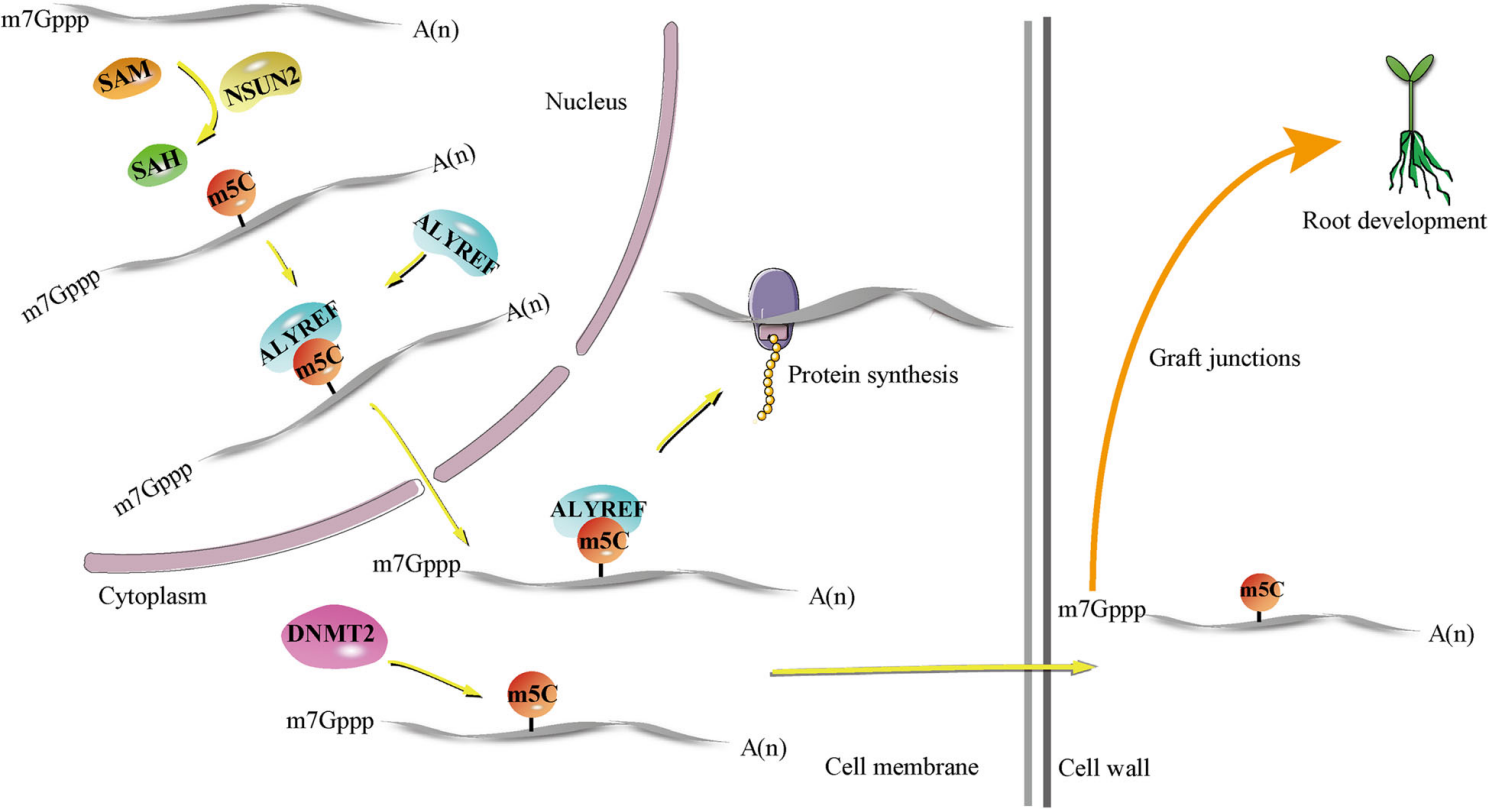
NSUN2-dependent m^5^C methylation in mRNA modulates nuclear-cytoplasmic export of the ALYREF-mRNA adduct. In plants, NSUN2- and DNMT2-induced m^5^C methylation facilitates mRNA transport to distant body parts over graft junctions, and proteins translated by methylated mRNA in target cells ultimately promote the root growth of plants

Cytosine methylation not only enables mRNA export from the nucleus to cytoplasm in human HeLa cells but also triggers mRNA transport to distant plant regions in *A. thaliana* (Fig. 2). Following bisulfite sequencing, m^5^C modification, which was found to be enriched in CG-rich regions as well as regions immediately after start codons, was confirmed to play a key role in mRNA nuclear-cytoplasmic shuttling [9]. Mechanistically, m^5^C methylation of mRNA was mediated by NSUN2, which increased the binding affinity of mRNA and ALYREF and ultimately modulated nuclear-cytoplasmic export of the ALYREF-mRNA binding adduct [9]. Moreover, m^5^C modification was also reported to be involved in transcript transport to regulate the development and growth of distant parts of plants. By generating mRNA methylation-deficient DNMT2-NSUN2 double mutants, researchers observed the diminished m^5^C modification and decreased mobility of graft-mobile transcripts, which ultimately inhibited root growth of *A. thaliana* [72].

## mRNA stability

Recent studies have also uncovered the significant role of m^5^C modification in affecting mRNA stability. It was reported in *A. thaliana* that m^5^C methylation modulated by TRM4B (also named NSUN2 in yeast and animals) plays an essential role in preventing the decay of root development-associated transcripts, thus maintaining mRNA stability and promoting plant growth [30]. Additionally, m^5^C modification is also involved in the early embryogenesis of zebrafish [69]. During the maternal-to-zygotic transition (MZT) of zebrafish, maternal mRNAs with m^5^C modification exhibit higher stability than unmodified mRNAs. This could be explained by the following mechanism: YBX1, together with its potential partner Pabpc 1a, specifically recognize and interact with m^5^C-modified mRNAs, thus maintaining maternal mRNA stability during MZT [69]. In contrast, some studies indicate that no correlation or a negative correlation was observed between the m^5^C level and mRNA stability [15, 72], and further investigations are urgently needed to clarify the molecular mechanisms of methylation-associated mRNA stabilization.

## tRNA

**Fig. 3:**
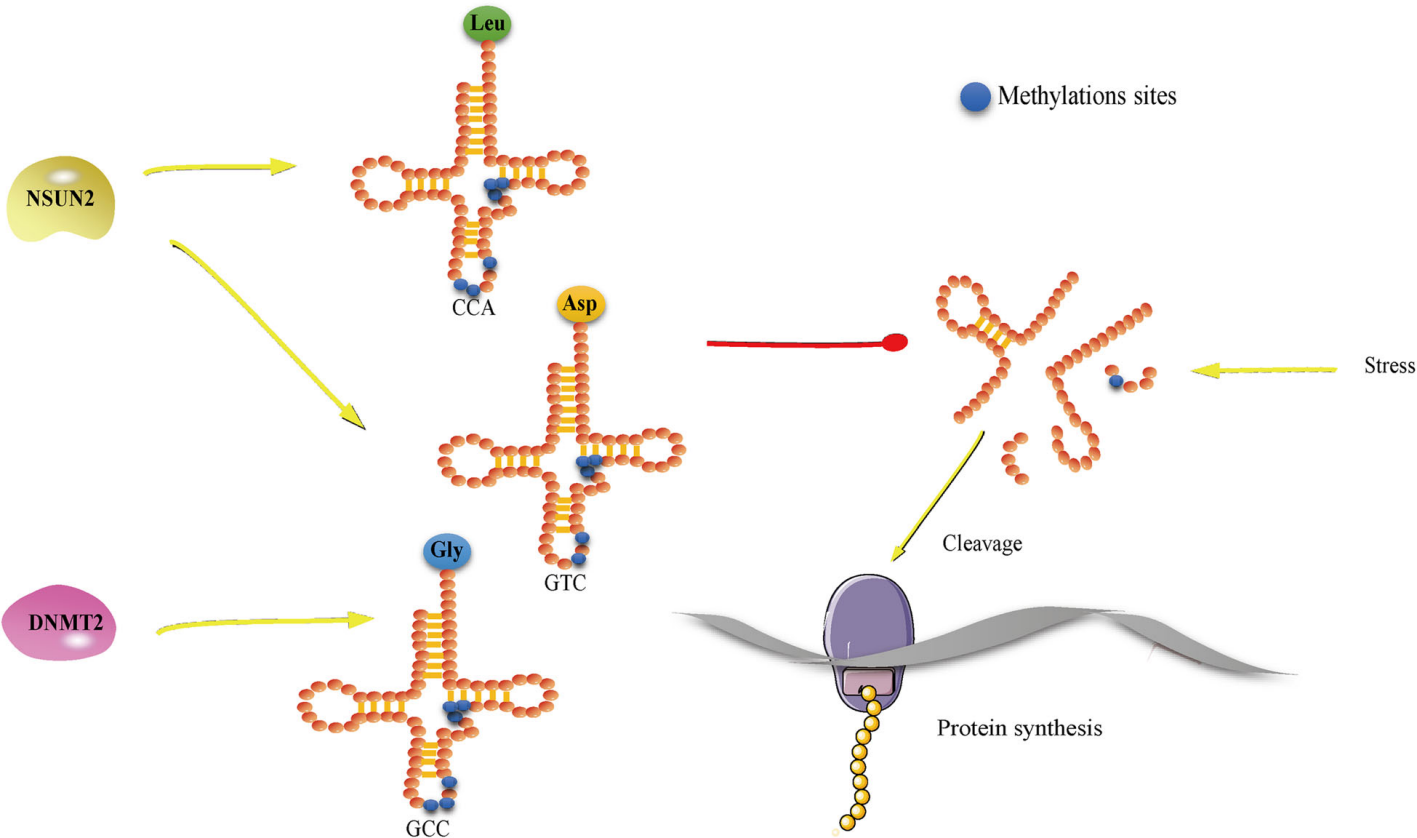
NSUN2-modified m^5^C methylation in tRNA^Leu-CCA^ maintains tRNA stability in response to stress and ultimately promotes protein synthesis. Additionally, DNMT2-dependent methylation of tRNA^Asp-GTC^ and tRNA^Gly-GCC^ protects them from cleavage, and this function is strengthened by NSUN2-modified m^5^C methylation of tRNA^Asp-GTC^ and tRNA^Gly-GCC^

tRNAs were the first identified substrates of RCMTs, with methylation sites mapped at positions C34, 38, 40, 48–50 and 72 within the anticodon loop. NSUN3 mediates cytosine methylation at position C34 of mitochondrial tRNAs, while NSUN6, NSUN2 and DNMT2 catalyze cytoplasmic tRNA methylation at the following positions: NSUN7-C72, DNMT2-C38, NSUN2-C34, 40, 48–49 [8, 22]. Notably, m^5^C modifications mediated by NSUN2 at position C48–50 in mt-tRNA have also been detected [42, 73]. tRNA m^5^C modifications mediated by NSUN2 and DNMT2 have been broadly demonstrated to maintain tRNA stability and regulate cell metabolism [15, 21, 22, 58, 72], as summarized in Fig. 3. In yeast, the proportion of TRM4/NSUN2-modified m^5^C sites in tRNA^Leu-CCA^ is increased to facilitate the translation of survival proteins in response to hydrogen peroxide, suggesting that reprogramming of tRNA modifications is required for the cellular stress response [73]. In humans, mice and plants, it was confirmed that m^5^C methylation by TRM4/NSUN2 prevents tRNA degradation from oxidative stress, and reduced tRNA stability and hypersensitivity to oxidative stress were observed in TRM4/NSUN2 mutants [15, 74]. Similarly, DNMT2-dependent methylation of tRNA protects it from endonucleolytic cleavage and modulates substrate tRNA^Asp-GTC^ and tRNA^Gly-GCC^ stability for accurate polypeptide synthesis during hematopoiesis [63]. Additionally, NSUN2 and DNMT2 were reported to cooperatively stabilize the common substrates tRNA^Asp-GTC^ and tRNA^Gly-GCC^, as double-knockout cells exhibited a robust reduction in abundance in comparison with either DNMT2−/− or NSUN2−/− single-knockout cells [39].

## rRNA

rRNA methylation can affect the structure of ribonucleoproteins and is essential for ribosome biogenesis. It was confirmed that Nop2/NSUN1 and Rcm1/NSUN5 mediates the methylation of 5-cytosine in domains V and IV of 25S rRNA in yeast [7] and that NSUN4 catalyzes the formation of m^5^C at position 911 of mammalian 12S rRNA [54]. The functional role of RCMTs in maintaining rRNA stability was also demonstrated to be similar to that in maintaining tRNA and mRNA stability. The loss of m^5^C2278, together with the loss of ribose methylation at G2288, resulted in a structural change in 25S RNA and the consequent loss of various ribosome proteins from the 60S large ribosomal subunit under low-salt conditions, thus impairing ribosome biogenesis [55]. Moreover, the effect of Rcm1/NSUN5 alone on ribosome conformation was also elucidated. When cells were exposed to hydrogen peroxide, the loss of Rcm1/NSUN5 led to a more relaxed 25S rRNA fold in the sequence near C2278, indicating that Rcm1/NSUN5 plays an essential role in maintaining rRNA stability in the presence of oxidative stress [56]. Metodiev et al. [75] proved that the m5C methyltransferase NSUN4 methylates cytosine 911 (m5C911) in small subunit (SSU) 12S rRNA and forms a complex with MTERF4 to ensure that only mature large subunits and SSUs are assembled into monomers. The above evidence proves that NSUN4 plays a key role in controlling the last step of ribosomal biogenesis.

## Other RNA species

Cytosine-5 methylation sites are also detected in viral RNAs; however, the regulatory role of m^5^C modification in viral RNAs is quite controversial. It was reported that DNMT2-mediated m^5^C methylation promotes HIV-1 genome RNA stability and ensures its survival in the host cell [65]. Moreover, NSUN2-dependent methylation of HIV-1 genomic RNA not only stimulates the translation of HIV-1 mRNA to produce proteins required for HIV-1 replication but also affects alternative HIV-1 RNA splicing [44]. In contrast, the adverse effects of m^5^C modification on viral RNA replication and stability have also been reported recently. NSUN1 catalyzes the methylation of HIV-1 TAR RNA at the 5′-UTR by competing with HIV-1 Tat protein, thus suppressing HIV-1 replication and facilitating its latency [53]. Additionally, by modulating m^5^C methylation of Epstein-Barr virus-encoded RNA 2 (EBER2), NSUN2 promotes RNase angiogenin-induced cleavage of EBER2 and leads to a decrease in its level [45]. Therefore, further studies are required to determine the reason different results were observed when investigating the role of m^5^C in viral RNAs.

Vault RNAs were originally derived from large ribonucleoprotein vault particles and participate in various biological processes [76]; NSUN2-catalyzed m^5^C was found in VTRNA1.1 and VTRNA1.3 [36]. NSUN2-mediated methylation of cytosine 69 stimulates the processing of VTRNA1.1 into small vault RNAs, thus promoting epidermal differentiation [47]. Cytosine-5 methylation is also enriched in lncRNAs and was demonstrated to modulate lncRNA functions [9, 15, 77]. The m^5^C methylation sites were mapped within or near the region mediating interaction with chromatin-modifying complexes in lncRNA HOTAIR and XIST and affected XIST function by influencing the binding of XIST to chromatin-associated protein complex PRC2 [78]. These results showed broad m5C profiles in diverse RNA species and highlighted their essential regulatory roles.

## The role of RNA m^5^C modification in human cancers

Since accumulating evidence has suggested the emerging role of RNA m^5^C modification in cancer pathogenesis and progression, below, we discuss the essential role of m^5^C modification as well as the underlying mechanisms in human cancers and propose m^5^C-based clinical approaches for cancer treatment.

## Bladder cancer

The RNA m^5^C modification landscape provides insights into dissecting the etiology of bladder cancer pathogenesis. It was reported that the overexpression of NSUN2 and YBX1 in cancer tissues promotes tumorigenesis by maintaining mRNA stability and is significantly correlated with poor prognosis in bladder cancer patients [23]. More specifically, the mRNA of the oncogenic gene heparin binding growth factor (HDGF) is methylated by NSUN2, with high enrichment in its 3′-UTR, and is then recognized by the RNA binding protein YBX1 [23]. By binding to m^5^C methylated sites and recruiting ELAVL1, YBX1 maintains HDGF mRNA stability and therefore exerts an oncogenic role in bladder cancer development through the activation of HDGF [23]. In addition, the enrichment of m^5^C mRNAs in tumor-related pathways, such as the PI3K-AKT [79] and ERK-MAPK signaling pathways [80], has also been observed, indicating that m^5^C hypermethylation might initiate tumorigenesis through the activation of oncogenic signaling pathways [69].

## Hepatocellular carcinoma (HCC)

As one of the most common malignances worldwide, HCC remains poorly understood in terms of its pathogenesis and development. Recently, the regulatory role of RNA m^5^C on HCC progression has been uncovered, and evidence has indicated that the elevated m^5^C regulators NSUN4 and ALYREF are negatively correlated with poor survival outcome in HCC patients and exhibit great value as significant diagnostic and prognostic biomarkers for HCC [24]. Using 6 pairs of HCC tissues and adjacent nontumor tissues, the same study group demonstrated that the number of mRNA m^5^C peaks in HCC tissues was significantly higher than that in adjacent tissues, with a more widespread distribution [81]. Together with the bioinformatics analysis results, the study group indicated that the alteration of m5C mRNA is correlated with the pathogenesis of HCC. However, the role of m^5^C regulators in cancer cell malignant phenotypes and the related molecular mechanisms remain to be further investigated.

## Glioblastoma multiforme (GBM)

miRNAs are also common m^5^C substrates, and the methylation of mature miR-181a-5p results in the abolishment of its tumor suppressor effect and is negatively correlated with poor prognosis in GBM. Mechanistically, mediated by the complex comprising DNMT3a and AGO4 (a miRNA-induced silencing complex), miR-181a-5p methylation impedes the formation of the miRNA-181a-5p/mRNA BIM duplex, which was originally defined to induce apoptosis by interacting with antiapoptotic Bcl-2 or Bcl-xl [14]. As a result, reduced apoptosis, as well as enhanced proliferation and invasion, was observed in cancer cells. In addition, as estimated by Kaplan-Meier analysis, the methylation level of miR-181a-5p is associated with a poor survival rate, suggesting the profound potential for attenuating m^5^C methylation and restoring normal miRNA function for cancer treatment [14].

## Leukemia

RNA m^5^C and RCMTs have been reported to participate in drug response/resistance not only in solid tumors but also in leukemia by interacting with distinct partners to regulate the formation of different chromatin structures. NSUN1 specifically interacts with BRD4 and then directly binds to RNA-pol-II CTD-S2P to form a unique NSUN1/BRD4/RNA-pol-II CTD-S2P complex in 5-azacitidine (5-AZA)-resistant leukemia cells, thus mediating the formation of a 5-AZA-resistant chromatin structure and inducing 5-AZA resistance in leukemia [18]. In contrast, NSUN3 and DNMT2 were confirmed to exhibit opposite effects on 5-AZA-sensitive leukemia cells [18]. Mechanistically, the RNA binding protein hnRNPK directly interacts with RCMTs (NSUN3 and DNMT2), lineage-determining transcription factors (GATA1 and SPI1/PU.1), and CDK9/P-TEFb and recruits RNA-polymerase-II at nascent RNA to form a distinct complex that results in the formation of a 5-AZA-sensitive chromatin structure [18]. Using clinical bone marrow specimens from 5-AZA-resistant and 5-AZA-sensitive leukemia cases, a clinical trial showed a significant increase in m^5^C mRNA in 5-AZA-resistant bone marrow compared with the 5-AZA-sensitive bone marrow. The expression levels of hnRNPK, NSUN1 and BRD4 were correlated with the course of leukemia and were involved in 5-AZA resistance and cancer development [18]. These results predict the potential role of m^5^C application in the treatment of hematologic malignancies.

Searching for efficient biomarkers for the diagnosis and prognosis of cancer and developing suitable targets for cancer treatment have always been the mainstream approaches against cancer. In HCC, it was confirmed that cancer tissues have higher RNA m^5^C levels than adjacent normal tissues [47]. Similarly, the number of mRNA m^5^C peaks was higher in drug-resistant cancer tissues than in drug-sensitive cancer tissues [18]. These results suggest that detecting the level of RNA m^5^C modification may facilitate cancer diagnosis. In addition, there is evidence suggesting the feasibility of using m^5^C and m^5^C regulators as prognostic biomarkers, which are critical for treatment planning and patient life expectancy [20, 23, 51]. Furthermore, as the elevated expression of RNA m^5^C regulators and the enrichment of m^5^C in RNA were correlated with the growth, proliferation, invasion, and drug resistance of cancer cells [18, 23, 69], targeting RNA m5C is available for developing new treatment strategies. However, although RNA m^5^C exhibits promising prospects in the diagnosis, prognosis and treatment of cancer, studies on RNA m^5^C are limited, and the cancer types studied are too few to construct a thorough review.

## Conclusion

In summary, existing evidence has revealed that RNA m^5^C methylation is essential for maintaining the normal physiological functions of cells and organisms, and its abnormal distribution and abundance are closely related to various diseases, including cancers. Therefore, it is proposed that RNA m^5^C methylation has great potential in the diagnosis, prognosis and treatment of cancer. Additionally, the mechanisms leading to the dysregulation of various RNA m^5^C regulators and the pathogenic mechanisms of RNA m^5^C methylation need to be further explored.

## Data Availability

Not applicable.

## Abbreviations

m^5^C: 5-methylcytosine
m^6^A: N6-methyladenosine
m^5^C-RIP-seq: m^5^C RNA immunoprecipitation sequencing
Aza-IP-seq: 5-azacytidine–mediated RNA immunoprecipitation sequencing
miCLIP-seq: methylation-individual nucleotide resolution crosslinking immunoprecipitation sequencing
RCMTs: respectively referring to RNA methyltransferases
TET: enzymes of ten-eleven translocation
HCC: hepatocellular carcinoma
GBM: glioblastoma multiforme
NGS: next-generation sequencing
Cs: cytosines
Us: uracils
Ts: thymines
SAM: S-Adenosyl methionine
5hmdC: 5-hydroxymethyl-2′-deoxycytidine
5fdC: 5-formyl-2’deoxycytidine
5cadC: 5-carboxyl-2′-deoxycytidine
YBX1: Y-box binding protein 1
CDS: cold-shock domain
UTR: untranslated region
EBER2: Epstein-Barr virus-encoded RNA 2
HDGF: heparin binding growth factor
